# Effect of legume proteins on the structure and digestibility of wheat starch-lauric acid complexes

**DOI:** 10.1016/j.fochx.2024.101891

**Published:** 2024-10-11

**Authors:** Jing Zhou, Shuang-yi Zheng, Qian-qian Chen, Xiao Wan, Jing Du, Wen-ping Ding, Xue-dong Wang, Hai-long Zhang

**Affiliations:** a Key Laboratory for Deep Processing of Major Grain and Oil, Ministry of Education, Wuhan Polytechnic University, Wuhan 430023, China; b Hubei Key Laboratory for Processing and Transformation of Agricultural Products, Wuhan Polytechnic University, Wuhan 430023, China; c National Engineering Research Center of Grain Storage and Logistics, Wuhan Polytechnic University, Wuhan 430023, China; d School of Food Science and Engineering, Wuhan Polytechnic University, Wuhan 430023, China

**Keywords:** Legume proteins, Starch-based complexes, Starch digestibility

## Abstract

The effect of legume proteins (soy protein (SP), chickpea protein (CP) and peanut protein (PP)) on the properties of wheat starch-lauric acid (WS-LA) system and its intrinsic mechanism were investigated. RVA, digestion experiment and TGA results showed that legume proteins prompted the viscosity peak formation during cooling stage and increased anti-digestion and thermal stability of WS-LA system. FT-IR, Raman, XRD and ^13^C NMR results indicated that legume proteins improved the long-range and short-range ordering degree and single-helix structure of WS-LA system. SP had greater influence on the properties of WS-LA system than that of CP and PP. Proteins with high solubility, emulsifying properties and β-sheet content were conducive to starch-based complexes formation. Molecular dynamics simulation results indicated that major forces for WS-LA-SP formation were hydrogen bonding and van der Waals forces. This study offered crucial information on starch-fatty acid-protein complexes formation for proteins selection in starch-based products development.

## Highlights

• Legume proteins improved order degree of starch in starch-lauric acid (LA) system.
• Soy protein (SP) was easier to form starch-LA-SP than chickpea and peanut protein.
• SP had greater effect on properties of WS-LA than chickpea and peanut protein.

## 1 Introduction

As one of the foremost carbohydrates in starch-based foods, starch determines the physicochemical, processing and nutritional properties of final processed products (Rostamabadi et al., 2023). The interactions of starch and other internal and external constituents of foods, for example fatty acids, proteins and phenolic compounds, can also influence these properties (Wang, Li, et al., 2023; Wang, Yu, et al., 2023). Specifically, the interaction of fatty acids and gelatinized starch mainly leached amylose changed the properties of starch by forming starch-fatty acid complexes (Liu et al., 2022). At present, starch-fatty acid complexes are drawing more and more attentions and designated as a new resistant starch (RS) (RS5) attributed to their lower digestion and remarkable prebiotic functions. Starch-fatty acid complexes have been reported to enhance intestinal health as well as decrease the probability of diet-associated diseases, for example obesity, diabetes and cardiovascular disease (Sun et al., 2023).

Some studies recently have found that proteins could complex with starch and fatty acids and have demonstrated starch-fatty acid-protein (SFAP) complexes formation during food processing (Lin et al., 2020). Starch-fatty acid complexes, protein-fatty acid complexes and protein aggregates linked by disulfide bonds have been considered as three possible structural elements of SFAP complexes (Lin et al., 2020; Parada & Santos, 2015). The main forces that form and stabilize SFAP complexes included various non-covalent interactions, for example, electrostatic interactions, van der Waals forces and hydrogen bonds, which result in greater alterations of starch physical and functional characteristics compared with starch-fatty acid complexes (Duan et al., 2023; Wang, Li, et al., 2023; Wang, Yu, et al., 2023). Specifically, SFAP complexes had higher anti-digestibility, solubility and ordered structure than starch-fatty acid complexes based on a previous study (Niu et al., 2020).

Several factors such as the characteristics and source of starch, protein and fatty acid and complexation conditions, influenced the formation of SFAP complexes. Protein, as the organizer, was supposed to be a crucial element for SFAP complexes formation. The structure and digestibility of SFAP complexes was influenced by protein type and whey protein isolate (isoelectric point lower than 7.0) was more favorable to the formation of rice starch-linoleic acid-protein complexes than A-type gelatin (isoelectric point higher than 7.0) based on an earlier study (Lin et al., 2020). Additionally, the emulsifying properties of protein was regarded as the most important properties influenced SFAP complexes formation attributed to the facilitation effect on the solubility of fatty acid (Duan et al., 2023). Legume proteins, such as peanut protein (PP), soy protein (SP) and chickpea protein (CP), as common plant protein sources with excellent emulsifying properties, are widely applied in food industry and nutritional fields. Therefore, legume proteins might be conducive to SFAP complexes formation by increasing the dispersion and solubility and of fatty acid. However, there was few studies reported on the effect of legume proteins on the digestibility and structure of WS-LA complexes. Additionally, the effect of others physical properties of protein on SFAP complexes formation was also unknown. Therefore, the effect of legume proteins including SP, PP and CP on the properties and formation of SFAP complexes and the intrinsic mechanism were studied using experimental method including rapid viscosity analysis (RVA), thermogravimetric analysis (TGA), fourier transformation infrared spectroscopy (FT-IR), X-ray diffraction (XRD), solid-state Nuclear Magnetic Resonance spectroscopy (^13^C NMR) and molecular dynamics simulation (MD). This report will offer a scientific basis for further development of anti-digestive starch-based complexes and promote the sustainable use and innovative development of plant-protein food products.

## 2 Materials and methods

### 2.1 Materials

Lauric acid (LA) with the purity of 98 %, 3,5-dinitrosalicylic acid (DNS), 8-anilino-1-naphthalenesulfonic acid (ANS) and wheat starch (WS) with 11.19 % moisture, 0.36 % ash, 0.45 % lipid, 0.32 % protein and 23.4 % amylose were offered by Shanghai Yuan ye Biological Co. Ltd. (Shanghai, China). α-Amylase (CAS: 8049-47-6) was offered by Sigma-Aldrich Co. LLC (Santa Clara, USA). Amyloglucosidase (CAS: 9032-08-0) was obtained from Shanghai Aladdin Biochemical Technology Co. Ltd. (Shanghai, China). BCA Protein Assay Kit was purchased by Wuhan Canos Technology Co. Ltd. (Wuhan, China).

### 2.2 Protein extraction

After blended with hexane (1:5 (*w*/*v*)), dehulled and pulverized beans were degreased in a magnetic stirrer (600 rpm) (SN-MS-6D, Shanghai Shangpu Instrument Co., Ltd., China) for 4 h at room temperature, and centrifuged (4000 r/min, 15 min, 4 °C). The above degreasing process was repeated for three times and the defatted beans were dried in a fume cupboard at room temperature. After blended with distilled water (1:10 (*w/v*)), the pH of dried defatted beans was adjusted to 8.0, 11 and 11 with 2 mol/L NaOH for soy bean and chickpea and peanut, respectively (Chen et al., 2024; González-Félix et al., 2023; Karaca et al., 2011). Then the above mixture was stirred (600 rpm) for 2 h at 25, 50 and 40 °C for soy bean, chickpea and peanut, separately. After centrifuged (8000 r/min, 15 min, 4 °C), the pH of the obtained supernatant was adjusted to 4.5 with 2 mol/L HCl. Subsequently, the above mixture was stand for 1 h and centrifuged (8000 r/min, 15 min, 4 °C) to obtain the target protein precipitate. After dissolving in distilled water, the pH value of the precipitation was adjusted to 7, dialysis (MWCO 8000–14,000 Da) was performed with deionized water at 4 °C for 48 h. The dialyzed samples were frozen at −20 °C for 24 h, and then freeze-dried at −40 °C (cold trap temperature) under a vacuum of 10 Pa for 48 h. SP (9.94 % moisture, 83.25 % protein and 3.90 % ash), CP (8.17 % moisture, 84.31 % protein and 3.58 % ash) and PP (10.21 % moisture, 82.99 % protein and 2.66 % ash) were collected, sieved through a 100-mesh sieve and used in subsequent experiments.

### 2.3 Starch-based complexes preparation

The preparation of starch-based complexes followed a method previously reported by Duan et al. (2023). The mixture (28 g) including WS (2.0 g), LA (0.1 g), protein (0.2 g) and appropriate distilled water was placed in a Rapid Viscosity Analyzer (RVA) (RVA-super 4, Perten, Sweden) and stirred manually with the plastic paddle before test. After equilibrated at 50 °C for 1 min, the temperature of the mixture was increased to 95 °C with a heating rate of 14 °C/min, and held at this temperature for 2.5 min. The temperature was then decreased to 50 °C with a cooling rate of 14 °C/min and held for 3.06 min. During the above process, the speed of the mixing paddle was 960 rpm for the first 10 s, then the speed of the mixing paddle was 160 rpm for the remainder of the experiment. The freeze-dried conditions of obtained samples were at −40 °C (cold trap temperature) under a vacuum of 10 Pa for 48 h. The freeze-dried sample was crushed and sieved with a sieve (100-mesh) for subsequent experiments including digestion, swelling power and solubility, TGA, complex index (CI), FT-IR, Raman, XRD and ^13^C NMR experiments.

### 2.4 In vitro digestion analysis

The digestion of sample was determined using the method of Englyst (Englyst & Hudson, 1996) with minor modifications. The sample (100 mg) was blended with acetic acid‑sodium acetate buffer (7.5 mL, pH 5.2, 0.1 mol/L) before pre-heated in a water bath (SHJ-6A, Changzhou Jintan Liangyou Instrument Co., Ltd., China) with a magnetic stirring speed of 300 rpm for 15 min at 37 °C. Subsequently, enzyme solution (2.5 mL, 16.5 U/mL amyloglucosidase and 290 U/mL porcine pancreatic α-amylase) was incorporated into the above solution and then maintained for 2 h at 37 °C in a magnetic stirring water bath (600 rpm). The digestive solution (0.1 mL) taken out at 0, 20, 40, 60, 80, 100, 120 and 180 min, respectively was blended with anhydrous ethanol (1.9 mL). Glucose content in the above solution was quantified by the external standard method of DNS. Rapidly digested starch (RDS), slowly digested starch (SDS) and resistant starch (RS) was obtained from Eqs. (1), (2), (3), respectively.

(1) $$\mathrm{RDS}\left(\%\right)=\left(G_{20}-G_{0}\;\right)/S\times 0.9\times 100$$

(2) $$\mathrm{SDS}\left(\%\right)=\left(G_{120}-G_{20}\right)/S\times 0.9\times 100\;$$

(3) $$\mathrm{RS}\left(\%\right)=\left(1-\mathrm{RDS}-\mathrm{SDS}\right)\times 100$$

where G_0_, G_20_, G_40_, G_60_, G_80_, G_100_, G_120_ and G_180_ were the mass of glucose (mg) in enzymatic solution after 0, 20, 40, 60, 80, 100, 120 and 180 min of enzymatic digestion, separately. S was total starch mass (mg) in sample.

### 2.5 Swelling power and solubility analysis

Solubility and swelling power of sample was measured using modified Cui's method (Cui et al., 2014). After dispersed in distilled water (3 mL), the sample (100 mg, m_1_) was magnetically stirred in a 95 °C water bath for 0.5 h, and centrifuged (4000 r/min, 20 min). After transferred to a constant weight aluminum box, the supernatant was dried at 105 °C to constant weight (m_2_). The weight of precipitate (m_3_) was recorded and the swelling power and solubility were calculated by Eqs. (4), (5) respectively.

(4) $$\text{Swelling power}\;\left(\%\right)=m_{3}/m_{1}\times 100$$

(5) $$\text{Solubility}\;\left(\%\right)=m_{2}/m_{1}\times 100\;$$

### 2.6 Thermogravimetric analysis

After sample (3 mg) was placed into a n alumina crucible, the thermal stability in the range of 30–700 °C was determined by a thermogravimetric analyzer (TGA-1100SF, Mettler Toledo, USA) under a condition of nitrogen with a heating rate of 10 °C/min.

### 2.7 Complex index analysis

After dispersed in 2.3 mL of distilled water, 0.2 g of sample was heated for 20 min at 100 °C. The obtained gel was blended with 12.5 mL of distilled water and then vortexed for 2 min and centrifuged (6000 r/min, 10 min). The mixture with 0.5 mL of supernatant, 15 mL of distilled water and 2 mL of KI solution was blended by vertexing. A_620 nm_ was measured and CI value was calculated using the Eq. (6).

(6) $$\mathrm{CI}\;\left(\%\right)=\left(A_{c}-A_{s}\right)/A_{c}\times 100$$

where A_C_ and A_S_ represented the absorbance of blank and sample, separately.

### 2.8 Fourier transformation infrared spectroscopy analysis

Before determination, the sample was blended with KBr (1:100 (*w/w*)), ground and compressed into transparent pellets. Infrared spectra in a range of 400–4000 cm^−1^ was determined by a FT-IR spectrometer (Frontier, Perkin Elmer Instruments Co., Ltd., USA) with a resolution of 4 cm^−1^ and scan number of 64. The spectra of all samples were treated by OMNIC 8.2 software (Version 8.2, Thermo-Nicolet Inc., USA) and deconvoluted with a resolution enhancement factor of 3.5 cm^−1^ and a half-band width of 4.0 cm^−1^.

### 2.9 Laser confocal micro-Raman spectroscopy

Raman spectra ranging from 3200 to 100 cm^−1^ were measured by Renishaw Micro-Raman Spectroscopy System (in Via-qontor, Renishaw, US) with a resolution of 7 cm^−1^. The full width at half-maximum (FWHM) of the band at 480 cm^−1^ obtained with software Origin 2021.

### 2.10 X-ray diffraction analysis

After equilibrium in saturated NaCl solution for 3 d, the sample were measured using a XRD diffractometer (Bruker D8, Bruker, Germany) with a Cu Kα X-ray source using continuous scanning conditions. XRD spectrum from 5° to 60° (2θ) were scanned at 5°/min and a tube pressure of 40 kV and a tube current of 40 mA. PeakFit v4.12 was used to calculate relative crystallinity (RC).

### 2.11 ^13^C CP/MAS NMR analysis

^13^C NMR spectroscopy were measured by a Bruker Avance Neo 400WB spectrometer (Bruker, Germany) at a resonance frequency of 100.63 MHz. After packed into a ZrO_2_ rotor with diameter of 4 mm, sample (0.2 g) was spun at the magic angle (54.7°) with 10 kHz of spin. The pulse width, contact time, acquisition time and delay were 6.00, 0.0157 s, 2 s and 1600 cycles, respectively. The obtained NMR spectra were peak-split by Peakfit v4.12 software to acquire single-helix, double-helix and amorphous components.

### 2.12 Properties and secondary structure of legume proteins

#### 2.12.1 Determination emulsifying properties of legume protein

The emulsifying properties of proteins was determined based on a previously reported method (Duan et al., 2023). After dissolved in 0.1 mmol/L Tris-HCl solution (9 mL, pH 8), protein (0.06 g) was vibrated in a shaker water bath at 25 °C for 30 min. Then soybean oil (3 mL) was added into the above solution, and then homogenized (10,000 r/min, 2 min). The emulsion of 50 μL was draw from the bottom of centrifuge tube and blended with 0.1 % SDS solution (5 mL) and then immediately vertexing for 5 s and A_0_ of the obtained solution was measured at 500 nm. After standing for 10 min, above procedures were repeated again and then the absorbance A_10_ was measured at 500 nm. The emulsifying activity index (EAI) and emulsifying stability index (ESI) of proteins was obtained according to the Eqs. (7), (8), separately.

(7) $$\mathrm{EAI}\left(m^{2}/g\right)=\frac{2\times 2.303\times A_{0}\times \mathrm{DF}}{C\times \Phi \times \left(1-\gamma \right)\times 10000}\;$$

where C was the concentration of protein solutions (g/mL), Φ was the optical range (1 cm), *γ* was the volume of oil in a homogeneous emulsion (0.25), and DF was the dilution factor; A_0_ was the absorbance value of sample at 0 min.

(8) $$\mathrm{ESI}\left(\min\right)=\frac{A_{0}}{A_{0}-A_{10}}\times 10\;$$

where A_0_, A_10_ was the absorbance value of sample at 0 and 10 min, separately.

#### 2.12.2 Determination of solubility of legume proteins

Protein solution (1 mg/mL) was prepared by blending protein (m_1_) with appropriate distilled water by vertexing for 30 s, and the above mixture was centrifuged (8000 r/min, 15 min, 4 °C). BCA protein assay kit was used to determine protein weight (m_0_) in the supernatant and the solubility of protein was calculated by Eq. (9).

(9) $$\text{Solubility}\;\left(\%\right)=m_{0}/m_{1}\times 100\;$$

#### 2.12.3 Secondary structure analysis of legume proteins

Protein secondary structure was determined using FT-IR as explained in the method of 2.8. To calculate the percentage of the secondary structure in legume proteins, the obtained infrared spectra were processed according to the reported method (Yasar et al., 2020).

### 2.13 Molecular dynamics simulations

Amylose used in MD simulations consist of 26 glucose units linked by α-1,4-glucoside bonds into an irregularly coiled single chain (Amy). Lauric acid (LA) was download from the PubChem database (CID number 3893) and 11S and 7S of soy protein were obtained from protein data bank (RCSB PDB). MD simulations were performed using Gromacs 2023.3 software package and a force field of Amber 14SB. TIP3P solvent model was used to solvate the ternary system. And the ternary system was encapsulated in a periodic cubic box 0.1 nm from the boundary and filled with water molecules, neutralized by adding antagonistic ions Na^+^/Cl^−^. The 50,000 steps of energy minimization under the force convergence criterion of 1,000 kJ/mol/nm were first performed using the step method, then NVT and NPT at 100 ps successively in the controlled temperature (310.15 K) and pressure were also conducted. V-rescale heat bath and Parrinello-Rahman pressure bath were selected to maintain desired temperature and the pressure (1 bar), respectively. The root means square deviation (RMSD), van der Waals (VDW), solvent-accessible surface area (SASA) and hydrogen bonding of starch-based complexes were determined using molecular mechanics Poisson-Boltzmann surface area method (MM-PBSA). Hydrogen bonds were measured by a maximum distance of 0.35 nm between the donor and acceptor atoms of hydrogen bond and an angle of less than 30° between bond donor and acceptor atoms. Visual molecular dynamics software was used to visualize the conformations at different time points.

### 2.14 Statistical analysis

SPSS 22.0 software (IBM, Armonk, NY, USA) with one-way analysis of variance (ANOVA) was used to statistically analyze the results. Duncan's test was used to determination the significant differences with a significance level of *P* < 0.05.

## 3 Results and discussion

### 3.1 Effect of legume proteins on the pasting properties of starch in WS-LA system

**Fig. 1:**
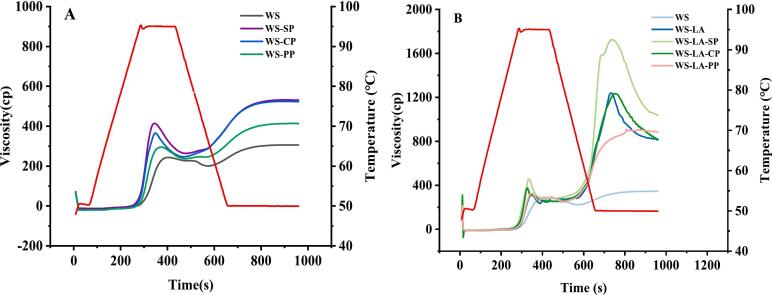
Effect of legume proteins on RVA profiles of WS (A) and WS-LA system (B).

As seen in Fig. 1 and Table 1, WS-protein all exhibited higher peak, setback and final viscosity than WS, especially for WS-SP and WS-CP. It might be due to many hydrophilic groups in SP and CP, which could crosslink with starch and increased water absorption of starch, resulting in the increase of starch swelling (Chinma et al., 2013). WS-LA also showed a higher peak, setback and final viscosity than WS, which might be due to WS-LA complexes formation (Chao et al., 2020). Compared with WS-LA, WS-LA-SP had significantly higher peak, setback and final viscosity, while WS-LA-CP and WS-LA-PP had no significant difference in peak, setback and final viscosity. This might be due to the better emulsifying properties of SP, which could form hydrogen bonds with starch to promote WS-LA or WS-LA-protein complexes formation (Cai, Li, et al., 2022; Cai, Tian, et al., 2022).

**Table 1:** Effect of legume proteins on peak viscosity, breakdown viscosity, setback viscosity and final viscosity of starch in WS-LA system.

Samples	Peak viscosity (cP)	Breakdown viscosity (cP)	Setback viscosity (cP)	Final viscosity
WS	273.65 ± 43.30^a^	50.88 ± 54.62^a^	121.88 ± 23.86^a^	344.63 ± 12.55^a^
WS-SP	401.00 ± 18.38^cd^	141.00 ± 12.73^e^	269.00 ± 2.83^b^	529.00 ± 2.83^b^
WS-CP	372.00 ± 8.49^b^^cd^	122.50 ± 4.95^de^	274.50 ± 2.12^b^	524.00 ± 1.41^b^
WS-PP	303.50 ± 12.02^ab^	64.00 ± 8.49^ab^	188.00 ± 16.97^a^	427.50 ± 20.51^ab^
WS-LA	319.50 ± 64.35^abc^	91.50 ± 21.21^cd^	585.00 ± 15.56^c^	813.00 ± 27.58^c^
WS-LA-SP	457.38 ± 14.67^d^	176.13 ± 0.35^f^	758.50 ± 19.09^d^	1039.75 ± 4.07^d^
WS-LA-CP	375.50 ± 45.96^bcd^	121.25 ± 88.21^de^	568.13 ± 66.64^c^	822.38 ± 24.40^c^
WS-LA-PP	327.75 ± 29.34^abc^	74.25 ± 79.37^ab^	632.63 ± 56.04^c^	886.13 ± 6.01^c^

Means with similar letters in the same column indicated that there was no significant difference (*p* > *0.05*).

The formation of cooling viscosity peak could be attributed to the rearrangement of starch-based complexes into more ordered structure during the cooling phase (Duan et al., 2023). There were no cooling viscosity peak in WS-protein systems, suggesting that no structurally ordered starch-based complexes were formed. WS-LA-SP had a significantly higher cooling viscosity peak compared with WS-LA and WS-LA-CP, whereas no cooling viscosity peak was observed for WS-LA-PP. This suggested that WS-LA-PP did not form starch-based complexes with orderly structure, or too few starch-based complexes were formed, which might be due to the poor emulsification performance of PP. It was similar to the result of Duan et al. (2023), who reported that poor emulsifying properties of egg white isolate proteins (EWI) resulted in slower formation of WS-palmitic acid-EWI.

### 3.2 Effect of legume proteins on the digestive properties of WS-LA system

**Fig. 2:**
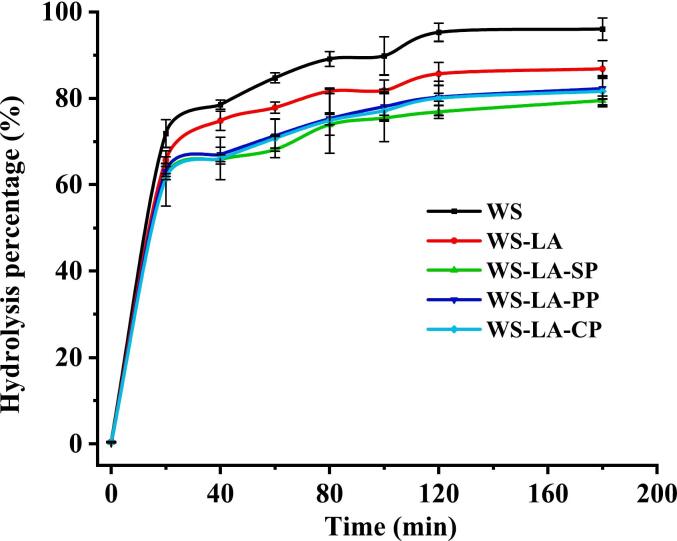
Effect of legume proteins on the enzymatic hydrolysis kinetics curves of WS-LA system.

The digestibility of all samples was risen rapidly within 20 min of enzymatic digestion and then increased slowly (Fig. 2). LA decreased the digestibility of WS at each digestion time, which was consistent with an earlier report (Zheng et al., 2018). Additionally, the anti-digestibility of WS-LA was furtherly decreased by proteins and SP had greater effect on the anti-digestibility of WS-LA system than CP and PP. As showed in Table 2, LA significantly enhanced RS content and reduced RDS content of WS. This was because LA, as a hydrophobic guest, inclined to entangle with starch to form WS-LA complexes, which decreased the contact of amylase with starch (Jia et al., 2018). Protein also furtherly enhanced RS content and declined RDS content of WS-LA system. This might be due to the formation of WS-LA-protein complexes with ordered structure and great steric hindrance, which reduced the contact between starch in WS-LA-protein complexes and amylase (Putseys et al., 2010). Additionally, legume proteins with a variety of hydrophilic groups and hydrophobic amino acids (proline, leucine, phenylalanine, valine, and isoleucine) could attach on the surface of starch through hydrogen bonding and/or hydrophobic interactions, which reduced the contact between amylase and starch (Chi et al., 2018). WS-LA-SP had higher RS content than WS-LA-PP, which might be due to the fact that SP formed more starch-based complexes with ordered structure than PP. Moreover, SP with high surface hydrophobicity and a flexible structure was easier to adhere to the surface of starch (Wu et al., 2023), while PP with tight structure had a weak binding to starch (Ochoa et al., 2017). The results was agreement with a previous study, which found that protein type significantly affected the formation of starch-based complexes with ordered structures, which in turn affected the digestibility of SFAP complexes (Lin et al., 2020).

**Table 2:** Effect of legume proteins on RDS, SDS and RS content and swelling power and solubility of starch in WS-LA system.

Samples	RDS%	SDS%	RS%	Swelling power%	Solubility%
WS	66.89 ± 0.73^a^	17.42 ± 0.61^a^	15.69 ± 0.12^a^	10.02 ± 0.15^a^	3.21 ± 0.07^ab^
WS-LA	63.88 ± 0.92^b^	18.47 ± 0.12^a^	17.65 ± 0.98^b^	9.44 ± 0.20^a^	2.08 ± 0.43^b^
WS-LA-SP	61.30 ± 0.76^c^	16.45 ± 0.06^a^	22.26 ± 0.81^d^	12.13 ± 0.87^b^	3.82 ± 1.04^a^
WS-LA-CP	60.71 ± 1.12^c^	18.63 ± 1.04^a^	20.66 ± 0.90^c^	11.87 ± 0.27^b^	3.62 ± 0.64^ab^
WS-LA-PP	62.78 ± 0.68^b^	16.93 ± 1.29^a^	20.28 ± 1.01^c^	11.20 ± 0.12^b^	2.58 ± 0.14^ab^

Means with similar letters in the same column indicated that there was no significant difference (*p* > 0.05).

### 3.3 Effect of legume proteins on solubility and swelling power of WS-LA complexes

Starch-based complexes formation affected the solubility and swelling power of starch in water, which were closely correlated with the processing characteristics of starch (De Pilli & Alessandrino, 2020). The solubility and swelling power of WS was slightly reduced by LA (Table 2), which might be because the un-complexed LA adhered to the surface of starch granules during heating and weakened the interaction between WS and water. This was consistent with the results of Du et al. (2023), who found that oleic acid significantly reduced the solubility and swelling power of WS. Compared with WS-LA, proteins were able to increase the solubility and swelling power of WS-LA system, which was in agreement with the findings of Wang et al. (2020). This might be attributed to WS-LA-protein formation, which reduced the un-complexed LA attached to WS, thus increasing the contact of WS and water.

### 3.4 Effect of legume proteins on the thermal properties of WS-LA system

**Fig. 3:**
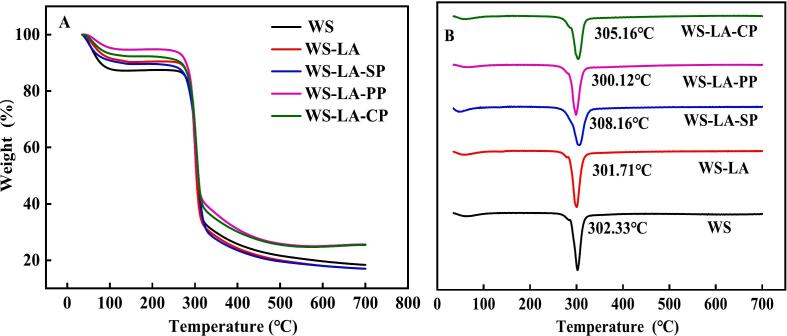
Effect of legume proteins on Thermogravimetric and micro-commercial thermogravimetric curves of WS-LA system.

As shown in Fig. 3A, two weight loss stages in samples appeared and the main weight loss in the first stage appeared at 30–150 °C, which was because the evaporation of water from starch-based samples (Chen et al., 2019; Gao et al., 2021). The weight loss in the first stage of WS-LA was lowered than that of WS, suggesting that LA increased the binding of starch and water. WS-LA-protein systems showed a lower weight loss in the first stage than WS-LA, implying that there was a synergistic effect between protein and LA on decreasing the weight loss of starch by prompting the binding between starch and water. The second stage of weight loss occurred at 250–380 °C, mainly due to thermal degradation of starch, fatty acids and proteins (Feng et al., 2024; Li et al., 2020). The thermal decomposition temperature of WS-LA had no significant increased compared with WS (Fig. 3B). SP and CP increased the thermal decomposition temperature of WS-LA system, which might be attributed to more V-type structural complexes formation, thus enhanced thermal stability of starch (Sun et al., 2021). Additionally, SP with flexible structure was more likely to expose hydrophilic groups and hydrophobic amino acids in thermal denaturation than PP with compact structure (Ochoa et al., 2017; Wu et al., 2023). Therefore, SP had greater effect on the improvement of starch thermal stability than PP due to the strong interaction between SP and starch.

### 3.5 Effect of legume proteins on the complexation index (CI) of WS-LA system

Legume proteins incorporation led to a significant increase in CI values of WS-LA (Table 3), which implied legume proteins prompted starch-based complexes formation or/and complexed with WS and LA. The CI value of WS-LA-SP was significantly greater than WS-LA-CP and WS-LA-PP. This might to be because high emulsifying properties of SP increased the solubility of LA in water allowing it to be better dispersed in the pasted starch and to have more contact with amylose, thereby facilitating starch-based complexes formation (Lin et al., 2020). Additionally, SP with a flexible structure was easily bound to the paste starch through hydrogen bonds between -OH groups from starches and the polar residual side chains or carbonyl groups from SP (Del Carmen Fernández-Alonso et al., 2012).

**Table 3:** Effect of legume proteins on complexation values (CI) and short-range ordered structure of starch in WS-LA system.

Samples	FWHM at 480 cm^−1^	1040/1022 cm^−1^	CI%
WS	19.25 ± 0.60^a^	0.44 ± 0.045^a^	–
WS-LA	18.37 ± 0.29^ab^	0.60 ± 0.083^ab^	55.22 ± 4.79^a^
WS-LA-SP	17.68 ± 0.60^b^	1.03 ± 0.29^c^	62.98 ± 0.50^c^
WS-LA-CP	18.25 ± 0.95^ab^	0.72 ± 0.035^b^	58.83 ± 0.98^b^
WS-LA-PP	18.56 ± 0.29^ab^	0.61 ± 0.067^ab^	59.12 ± 0.78^b^

Means with similar letters in the same column indicated that there was no significant difference (*p* > 0.05).

### 3.6 Effect of legume proteins on the short-range ordered structure of WS-LA system

**Fig. 4:**
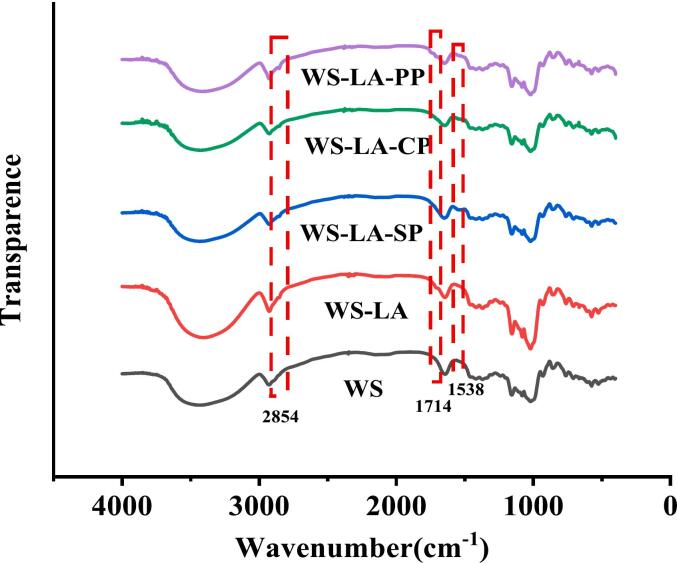
Effect of legume proteins on FT-IR spectra of WS-LA system.

As shown in Fig. 4, compared with WS, two pronounced new absorption peaks of WS-LA appeared at 2850 and 1740 cm^−1^, which were due to the stretching vibration of —C—H— and —C=O— on the aliphatic chain of LA molecule (Wang et al., 2017). While the absorption band at 1740 cm^−1^ was disappeared in WS-LA-protein system, which might be attributed to WS-LA-protein complexes formation attenuating the IR absorbance of carbonyl group from LA. The ratio of peaks intensity at 1040 cm^−1^ and 1022 cm^−1^ could represent the amount of ordered crystals in starch relative to the amorphous regions, and starch with large of 1040/1022 cm^−1^ value suggested that it had high short-range ordering degree (Liu et al., 2020; Van Soest et al., 1994; Wang et al., 2016). LA had no significant effect on 1040/1022 cm^−1^ value of WS, while SP and CP significantly increased 1040/1022 cm^−1^ value of WS-LA (Table 3). This might be due to the high emulsifying properties of SP and CP, which enhanced the arrangement of WS-LA complex into a more ordered and crystalline structure (Lin et al., 2020).

**Fig. 5:**
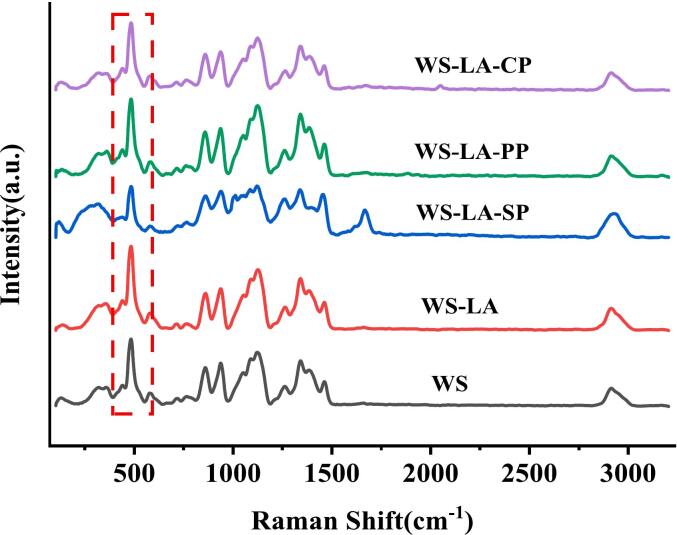
Effect of legume proteins on Raman spectra of WS-LA system.

The half-width at maximum (FWHM) of Raman band of starch at 480 cm^−1^ was lower, indicating that the degree of short-range ordering of starch was high (Marinopoulou et al., 2016). LA reduced the FWHM value of WS by 4.6 % (Table 3 and Fig. 5), suggesting that LA increased the short-range ordered structure of WS might ascribed to starch-based complexes formation. The FWHM value of WS-LA was furtherly reduced by 3.8 % due to SP incorporation, indicating SP increased the degree of short-range ordering of starch in WS-LA system. SP had greater effect on increasing the degree of short-range ordering of WS in WS-LA system than CP and PP, which was in agreement with FT-IR result.

### 3.7 Effect of legume proteins on the long-range ordered structure of WS-LA system

**Fig. 6:**
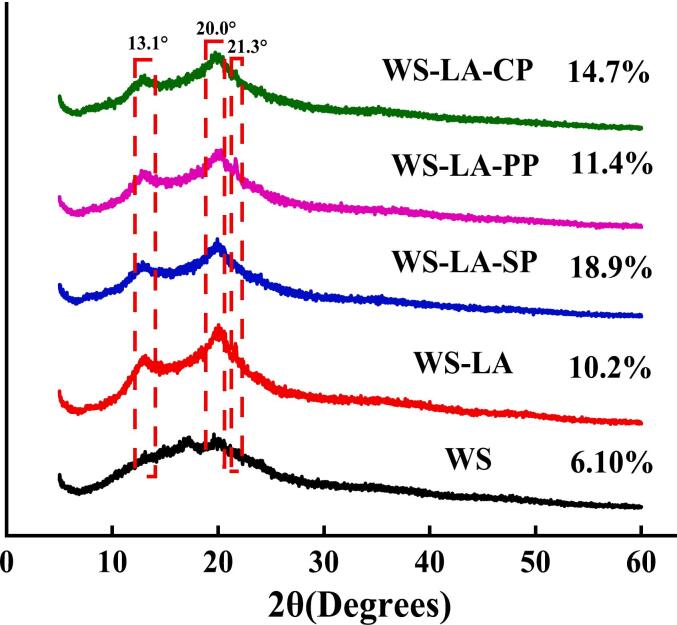
Effect of legume proteins on XRD patterns of WS-LA system.

The diffraction peaks at 13.1° and 20.0° were appointed as the typical V-type starch-based complexes, and the diffraction peak at 21.3° was free fatty acid (Cai, Li, et al., 2022; Cai, Tian, et al., 2022; Yang et al., 2020). WS showed a weak diffraction peak at 20.0°, while WS-LA displayed two strong diffraction peaks at 13.1° and 20.0° and a weaker diffraction peak at 21.3° (Fig. 6). Additionally, WS-LA-SP and WS-LA-CP also had two strong diffraction peaks at 13.1° and 20.0°, and a weaker diffraction peak at 21.3°, suggesting there was a V-type starch-based complex formation. While WS-LA-PP had two weak diffraction peaks at 13.1° and 20.0° and a strong diffraction peak at 21.3°. It was consistent with an earlier findings of Zheng et al. (2018), who attributed the diffraction peak at 21.3°(2θ) to the aggregation of un-complexed fatty acids. LA increased the crystallinity of WS, and protein furtherly increased the crystallinity of WS-LA system, suggesting that LA and protein had a promoting effect on the V-type complexes formation. WS-LA-SP showed highest crystallinity and WS-LA-PP showed lowest crystallinity among WS-LA-protein system.

### 3.8 Effect of legume proteins on the helical structure of starch in WS-LA system

**Fig. 7:**
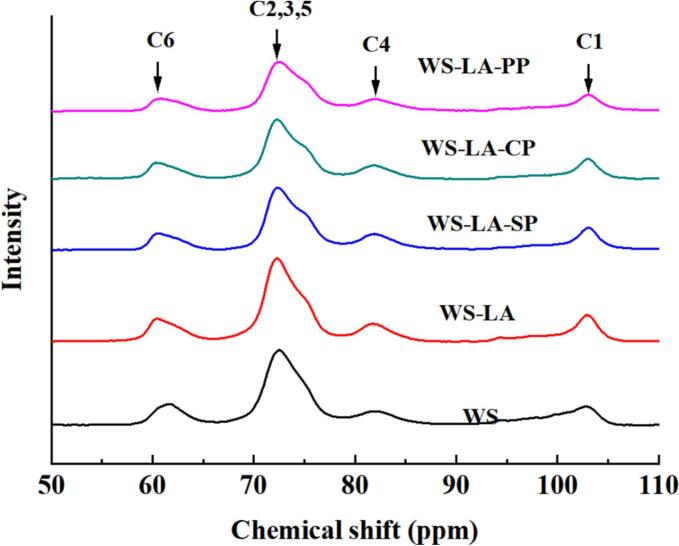
Effect of legume proteins on Solid-state ^13^C NMR spectra of WS-LA system.

The chemical shifts of the peaks in solid-state ^13^C NMR spectra could characterize helix structure of starch (Nonappa & Kolehmainen, 2016). As shown in Fig. 7, there were four characteristic peaks at 100–110, 70–79, 80–84, and 58–65 ppm, which was equivalent to C1, C2,3,5, C4 and C6, respectively. The peak at 103 ppm in the crystalline region might be due to the small amount of V-type single helix structure presented in starch (Thérien-Aubin et al., 2007). The relative content of V-type single helix, double helix and amorphous structures was obtained by calculating the ratio of the peak area of amorphous and crystalline regions to the total area of sample (Tan et al., 2007). As shown in Table 4, LA increased the helix structure content including double-helix and single-helix structure of WS, which ascribed to the transformation of amorphous into helix structure due to the complexation of amorphous amylose and LA. Protein furtherly increased the helix structure content including single-helix and double-helix structure of WS-LA system. This observation was consistent with the change in setback viscosity of WS-LA-protein system (Table 1), suggesting that legume proteins prompt the formation of starch-based complex with high helical structures by influencing starch reassembly during cooling stage (Lin et al., 2020). Additionally, the helix structure content of WS-LA-SP system was obviously greater than that of WS-LA-CP and WS-LA-PP. Form the results of the effect of legume proteins on the structure of starch in WS-LA system, it could conclude that SP had greater effect on the short-range and long-range ordered structure, and helical structure of starch in WS-LA system than CP and PP. It might be due to the better emulsifying properties and higher hydrophobicity of SP were more favorable for the formation of V-type starch-based complexes with high ordered structure (Wang et al., 2017).

**Table 4:** Effect of legume proteins on amorphous, single-helical and double-helical content of starch in WS-LA system.

Samples	Amorphous content/%	Single-helix content/%	Double-helix content/%
WS	53.74	1.72	44.54
WS-LA	44.72	4.09	51.18
WS-LA-SP	23.82	11.58	64.60
WS-LA-CP	42.38	6.70	50.91
WS-LA-PP	42.98	5.15	51.88

### 3.9 The properties and secondary structure of legume proteins and the correlation of it with the structure of starch in WS-LA-protein system

The emulsifying properties of protein was thought to be a key factor in promoting the formation of SFAP complexes (Wang et al., 2017). EAI value of SP was significantly greater than that of CP and PP and ESI value of SP and CP had no significant difference and were significantly greater than that of PP (Table 5). Therefore, emulsifying properties of the three legume proteins were SP, CP and PP in descending order. The solubility of proteins can reflect the hydration ability between proteins and water molecules, protein with high solubility indicates that the protein with strong binding ability between water molecules and proteins, which is conducive to the function of protein (Li et al., 2020). The solubility of SP was significantly greater than that of CP and PP, which might be related to the higher content of hydrophilic amino acids in SP (Cai, Li, et al., 2022; Cai, Tian, et al., 2022). It was clear that SP was more easily to form the complexes by complexing with WS and LA, while PP was difficult to form complexes with WS and LA in the present study. As shown in Table 6, the emulsifying properties, solubility and β-sheet content of legume proteins displayed a positive correlation with the short-range/long-range ordered structure. Therefore, it was believed that solubility, emulsifying properties and β-sheet content influenced starch-based complexes formation.

**Table 5:** Protein content and emulsification characteristics, solubility and β-sheet content of legume proteins.

Samples	EAI (m^2^/g)	ESI (%)	Solubility (%)	β-sheet (%)
SP	6.49 ± 0.01^a^	7.62 ± 0.23^a^	32.41 ± 2.53^a^	26.77 ± 0.55^a^
CP	4.53 ± 0.01^b^	7.90 ± 0.02^a^	13.82 ± 0.13^b^	28.20 ± 0.16^a^
PP	2.64 ± 0.01^c^	6.79 ± 0.09^b^	10.68 ± 2.00^b^	35.83 ± 2.92^b^

Means with similar letters in the same column indicated that there was no significant difference (*p* > 0.05).

**Table 6:** Correlation between amplifiability, solubility, β-sheet content, FWHM, 1040/1022 cm^−1^, RS and single-helix content.

	Emulsifiable	Solubility	β-sheet	FWHM	1040/1022 cm^−1^	Crystallinity
Emulsifiable	1					
Solubility	0.929	1				
β-sheet	−0.926	−0.721	1			
FWHM	−0.987	−0.976	0.855	1		
1040/1022 cm^−1^	0.967	0.993	−0.799	−0.995	1	
Crystallinity	0.998*	0.949*	−0.902	−0.995	0.980	1

* and ** indicated the significant difference at *P < 0.05* and *P < 0.01* level, respectively.

### 3.10 The interaction force of WS-LA-SP complexes

**Fig. 8:**
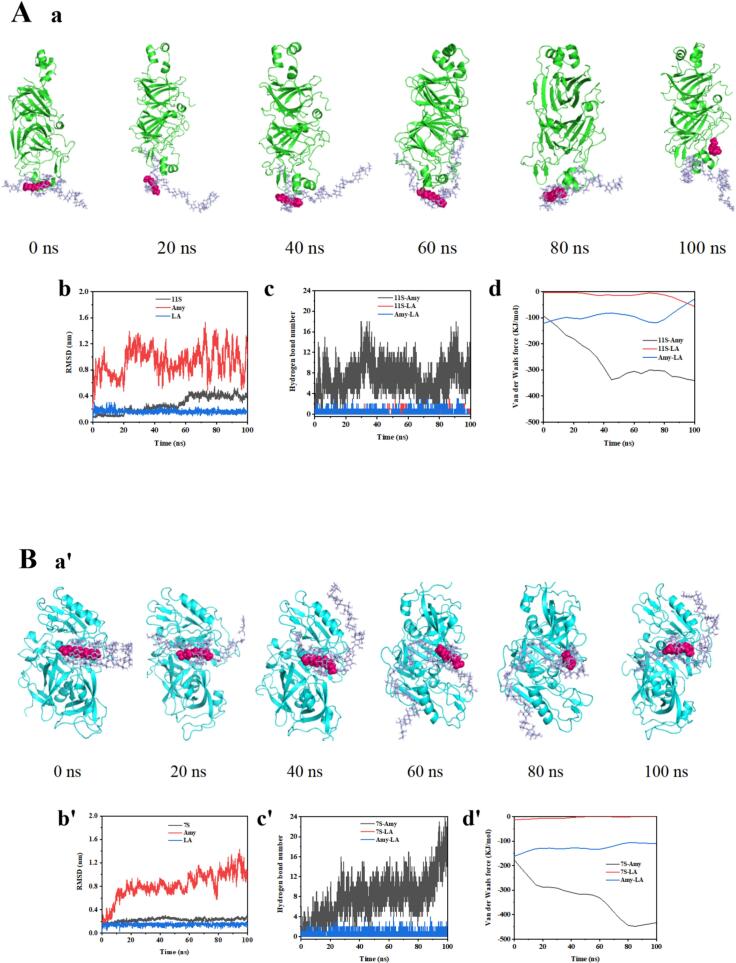
MD simulations of Amy-LA-11S (A) and Amy-LA-7S complexes (B) formation. Snapshots of conformational changes (a), RMSD (b), hydrogen bonding number (c), and van der Waals forces (d) for Amy-LA-11S complex. Snapshots of conformational changes (a'), RMSD (b'), hydrogen bonding number (c') and van der Waals forces (d') of Amy-LA-7S complex.

These results revealed that SP was easier to form starch-lipid-protein complexes than CP and PP in this study. To better understand the mechanism of WS-LA-SP formation, MD simulations were performed using amylose containing 26 glucose residues, LA and 11S (glycinin) or 7S (β-conglycinin), primary constituents of soy protein (Ye et al., 2023). The conformational changes of Amy-LA-11S and Amy-LA-7S at different times were shown in Fig. 8Aa, Ba'. Compared with 11S, the binding of 7S to starch and LA was stronger because Amy-LA-7S system was still firmly adsorbed on the surface of 7S at the end of simulation and Amy-LA-11S system finally depolymerized. From the results of RMSD values (Fig. 8Ab, Bb’), 11S and 7S remained relatively stable during simulation, whereas amylose had greater changes in Amy-LA-11S system than in Amy-LA-7S system. From the results of hydrogen bonding (Fig. 8Ac, Bc’) and van der Waals force (Fig. 8Ad, Bd’) analysis, it could be seen that van der Waals forces and hydrogen bonds formed in Amy-LA-7S system were more and stronger than that in Amy-LA-11S system during Amy-LA-protein complexes formation. It was speculated that the conformation of Amy-LA-7S was more stable than Amy-LA-11S, which might be ascribed to more hydrogen bonding and stronger van der Waals forces existed in Amy-LA-7S system and was consistent with an earlier study (Wang, Li, et al., 2023; Wang, Yu, et al., 2023). From the result of free binding energy (Table 7), Amy-protein interaction gradually increased during simulation process and the interaction between amylose and 7S was greater than that between amylose and 11S. Additionally, the forces existed in Amy-LA-7S system were stronger than that in Amy-LA-11S system, which might be that the incorporation of 7S resulted in an alteration of starch structure and thus promoted the binding of LA to starch. Therefore, 7S was the main components altered the physical properties and structure of starch in WS-LA system.

**Table 7:** Interacting forces and average energy parameters for Amy-LA-11S and Amy-LA-7S system obtained from 0 to 100 ns of MD simulation.

System		Electrostatic (kJ/mol)	MM potential (kJ/mol)	Polar	SASA	Solvation (kJ/mol)	Binding free energy (kJ/mol)
Amy-LA-11S	11S-Amy	−141.94	−376.38	380.30	−31.16	348.68	−27.70
	11S-LA	−3.48	−17.06	4.29	−2.55	1.60	−15.46
	Amy-LA	−6.42	−96.81	50.23	−11.60	38.70	−58.11
Amy-LA-7S	7S-Amy	−59.48	−195.60	175.24	−19.54	155.51	−40.09
	7S-LA	−0.29	−4.040	4.31	−44.00	3.68	−0.36
	Amy-LA	−13.37	−116.94	58.91	−11.67	46.46	−70.48

## 4 Conclusions

Legume proteins including SP, CP and PP promoted starch-based complexes formation to varying degrees, and increased the ordered structure and decreased digestibility of starch in WS-LA system. SP was more readily to form WS-LA-protein complexes than CP and PP attributed to the better emulsifying properties and solubility of SP, which solubilized LA more readily than CP and PP, thereby facilitating ternary interactions. The emulsifying properties, solubility and β-sheet content of legume proteins were positively correlated with the short-range/long-range ordered structure of starch in WS-LA system and were thought to the main factors influencing starch-based complexes formation. Hydrogen bonding and van der Waals forces were main force of WS-LA-SP complexes. While only three common legume proteins used in this research, more proteins with different structures and functions deserve further investigation in future studies.

## CRediT authorship contribution statement

**Jing Zhou:** Investigation, Methodology, Software, Manuscript revision, Writing-Original Draft, Writing –review & editing. **Shuang-yi Zheng:** Methodology, Investigation, Data curation. **Qian-qian Chen:** Resources, Investigation, Data curation. **Xiao Wan:** Methodology, Investigation, Data curation. **Jing Du:** Writing – original draft, Project administration, Methodology, Investigation, Conceptualization. **Wen-ping Ding:** Methodology, Formal analysis, Data curation. **Xue-dong Wang:** Resources, Project administration, Investigation, Funding acquisition. **Hai-long Zhang:** Writing – original draft, Resources, Project administration, Investigation, Funding acquisition, Conceptualization.

## Declaration of competing interest

The authors declare that they have no known competing financial interests or personal relationships that could have appeared to influence the work reported in this paper.

## Data Availability

No data was used for the research described in the article.
